# Analysis of Complications and Risk Factors Other than Bleeding before and after Endoscopic Treatment of Esophagogastric Variceal Bleeding in Patients with Liver Cirrhosis

**DOI:** 10.1155/2023/7556408

**Published:** 2023-03-29

**Authors:** Xiaowei Duan, Xing He, Hezhong Yan, Haiqing Li, Jiaoxue Wang, Shicun Guo, Zhengwei Zha, Qianqian Zhang, Yuchuan Bai, Jiayi Zhang, Jun Tang, Derun Kong

**Affiliations:** ^1^Department of Gastroenterology, The First Affiliated Hospital of Anhui Medical University, Hefei, Anhui 230022, China; ^2^Department of Gastroenterology, The 901 Hospital of Joint Logistics Support Force, Hefei, Anhui 230031, China; ^3^Department of Gastroenterology, First People's Hospital of Hefei, Hefei, Anhui 230031, China

## Abstract

**Objective:**

To identify any concomitant complications other than bleeding (COTB) before and after endoscopic treatment of esophagogastric variceal bleeding (EGVB) in liver cirrhosis patients and explore the underlying risk factors.

**Materials and Methods:**

Cirrhotic patients complicated with EGVB, who underwent interventional endoscopic treatments in our hospital from November 2017 to August 2020, were enrolled in this study. Clinical data were retrospectively analyzed for COTB at admission and within 2 years of the first endoscopic treatment. Patients were screened for potential risk factors of COTB before and after the treatment. Univariate analysis was performed to identify clinical factors of secondary complications, and statistically significant factors were included in the multivariate Cox and logistic regression analyses.

**Results:**

Of the 547 patients with cirrhosis, 361 individuals had COTB in the first endoscopic treatment. In this cohort, the top 3 prevalent incidences were portal vein thrombosis (PVT) or spongiosis, cholelithiasis, and pathogenic infections. The COTB did not occur at admission in 171 liver cirrhosis patients but happened at the follow-up. Higher Child-Pugh scores indicated potential risks of multiple concurrent complications, including bleeding. Risk factors for concomitant PVT or cavernous changes after endoscopic treatment of EGVB, pathogenic infections, and cholelithiasis could prolong the cirrhosis symptoms, while noncholestatic cirrhosis patients might have a lower risk than posthepatitis B cirrhosis patients, in the context of a higher degree of EGV and serum level of D-D and a lower blood calcium level.

**Conclusions:**

Clinical treatment and interventions can be tailored to avoid other complications during and after EGVB treatment, which can affect the outcome and prognosis of bleeding symptoms.

## 1. Introduction

Cirrhosis in the liver is an advanced-stage liver injury characterized by multiple severe scars and compromised liver function involving multifactorial etiologies. However, in Europe and the United States, the major etiology is alcoholic cirrhosis, accounting for increasing trends in liver disease-related morbidity and mortality [1]. Notably, the incidence rate of viral hepatitis-induced liver cirrhosis is significantly higher in the Chinese population than that in Western countries. Undoubtedly, liver cirrhosis has become one of the leading public health issues globally [2]. Although an early stage of liver cirrhosis can be cured, however, the later stage of the disease can lead to a spectrum of secondary complications, including progressive portal hypertension, systemic inflammation, liver failure, liver encephalopathy, and primary liver cancer [3], resulting in poor treatment outcomes [4]. Interestingly, some patients develop complications sequentially, while others may develop multiple secondary symptoms simultaneously. Notably, the most common, aggressive, and fatal complication in liver cirrhosis patients is the recurrent incidences of esophagogastric variceal bleeding (EGVB) [5–7]. The mortality rate of cirrhosis complicated by upper gastrointestinal bleeding is more than 40% [8]. EGVB symptoms are usually treated with endoscopic ligation or injection of sclerosing agents [9, 10], which may have adverse contraindications, and the treatment efficacy is largely related to the overall health condition of the patient [11]. It has been reported that the failure rate of EGVB treatment can be as high as 20% [12]. Moreover, any underlying comorbidity such as ascites, infections, portal thrombosis, and electrolyte imbalances can negatively impact the outcome of endoscopic treatments and prognosis in liver cirrhosis patients [13–15]. Importantly, even after successful treatment, advanced-stage patients can recur an aggressive form of EGVB during the follow-up period [16, 17]. Therefore, we designed this study to retrospectively analyze the clinical data of 547 liver cirrhosis patients who were treated endoscopically for EGVB and reported recurring symptoms after 2 years of the treatment towards identifying biomarkers that might facilitate the early diagnosis and improved interventional therapies. Furthermore, we analyzed the risk factors for the occurrence of complications other than bleeding at the time of admission and follow-up as well.

## 2. Patients and Methods

### 2.1. Study Population

The study population consisted of patients admitted to the First Affiliated Hospital of Anhui Medical University, Hefei, China, from November 2017 to August 2020 for initial endoscopic treatment of cirrhosis-associated EGVB, including ligation, sclerosis, tissue glue, and combined multimodal therapies. The following criteria were implemented to enroll study subjects: (1) diagnosed with cirrhosis and EGVB according to the Bevano VI criteria [18, 19] and (2) having complete clinical data. While the exclusion criteria included the followings: (1) presenting with excessive bleeding leading to systemic shock or even death, (2) coagulopathy, (3) cirrhotic gastrointestinal bleeding other than a variceal rupture, and (4) presenting comorbidities involving other systemic malignancies (except hepatocellular carcinoma, HCC) [20]. The data analyzed were extracted from a retrospectively collected database, including patients' characteristics, laboratory indices, imaging examinations, and endoscopic diagnoses.

### 2.2. Data Collection

Clinical information of non-EGVB cirrhotic complications [21], including gallbladder stones, infections, portal vein thrombosis (PVT) or spongiosis, liver encephalopathy, electrolyte imbalance, hepatorenal syndromes, hepatopulmonary syndromes, and primary HCC were extracted from the patient records at admission and within 2 years of the first endoscopic treatment.

A total of 50 general clinical variables and physicochemical indicators, including sex, age, time to detect cirrhosis, body mass index (BMI), blood pressure (BP), heart rate (HR), presence of liver palms/spider nevus, history of splenectomy, education level, smoking, alcohol consumption, antiviral drug use, treatment with beta blockers, cause of cirrhosis, diabetes mellitus, hepatitis B virus titer, degree of EGV and its treatment, the Child-Pugh score for cirrhosis mortality, concomitant HCC, presence of portal hypertensive gastropathy, as well as the following biochemical indices: D-dimer (D-D), international normalized ratio, prothrombin time, serum levels of alanine aminotransferase (ALT), aspartate aminotransferase (AST), aspartate aminotransferase (AST), albumin (Alb), gamma-glutamine transferase (GGT), alkaline phosphatase (ALP), total bilirubin (TB), cholinesterase (ChE), creatinine (Cr), urea, glomerular filtration rate (GFR), triglycerides (TG), cholesterol, serum calcium (sCa^+2^), serum sodium (sNa^+^), neutrophil (Neu) and lymphocyte count, neutrophil-to-lymphocyte ratio (NLR), white blood cell (WBC) count, hemoglobin (Hb), platelet (PLT) count, red blood cell (RBC) distribution width, alpha-fetoprotein (AFP), carcinoembryonic antigen (CEA), peritoneal fluid, and CLIF consortium acute decompensation (CLIF-C AD) score, were retrieved, reviewed, and documented at the admission.

### 2.3. Observational Indicators

Univariate logistic regression analysis was performed using 50 clinical and physicochemical indicators of cirrhosis-associated complications other than EGVB at admission and within 2 years of the first endoscopic treatment. Variables with statistically significant differences in the univariate analysis were subjected to a multivariate logistic or Cox regression analysis to detect any risk factors for concurrent and sequential COTB.

### 2.4. Statistical Analysis

IBM SPSS Statistics for Windows, version 25 (IBM Corp., Armonk, NY, USA), was used for data entry and statistical analysis. Measurement data were represented as mean ± standard deviation (SD), and the independent samples *t*-test was used for comparing the groups. Count data were described as the rate (percentage) and the 2-test was used for between-group comparisons. One-way and multiway logistic regression analyses were performed to examine the risk factors affecting patients with cirrhotic bleeding with other complications. While one-way and multiway Cox regression analyses were used for assessing risk factors affecting patients with cirrhotic bleeding at a later stage. A *p* value of <0.05 was considered statistically significant.

## 3. Results

### 3.1. Patients' Characteristics

In this study, a total of 547 patients, including 375 men and 172 women, with cirrhotic EGVB were enrolled. The age range of included patients was 18–91 (54.76 ± 11.89) years. The time to discovery of cirrhosis was 0–420 months (median: 24, and interquartile range (IQR): 5, 72). The etiological classification of posthepatitis B cirrhosis was identified in 280 (51%) subjects; of these, 124 patients were male and 47 were female presenting other complications at the follow-up.

### 3.2. Diagnosis of EGVB-Associated COTB at Admission and Follow-Ups

Among the 547 patients, 361 patients presented other complications of cirrhosis at admission, excluding EGVB. In addition to bleeding, the top three most prevalent incidences were PVT and/or cavernous lesions (*n* = 178), cholelithiasis (*n* = 152), and infections (*n* = 101). Interestingly, some patients had a combination of 2 or more complications.

Of the 547 patients, ligature treatment was performed in 251 subjects, sclerotherapy in 136 cases, tissue glue in 18 cases, and combined treatment in 131 cases. A total of 171 cases of COTB occurred during the follow-up observations, and the top three most prevalent complications in terms of incidence rate were PVT or spongiosis (*n* = 89), infection (*n* = 70), and cholelithiasis (*n* = 68), including some patients with ≥2 complications at the same time. Moreover, infection-associated complications could be subdivided into the following categories: spontaneous peritonitis (*n* = 20), biliary tract infection (*n* = 1), pulmonary infection (*n* = 37), intestinal infection (*n* = 3), urinary tract infection (*n* = 9), and spontaneous peritonitis cum pulmonary infection (*n* = 7), indicating that the pulmonary infection was the highest (53%), followed by spontaneous peritonitis (29%) among all the prevailing complications.

The top three complications of cirrhosis at admission and follow-up were PVT and/or cavernous changes, cholelithiasis, and infections, except for EGVB (Table 1).

### 3.3. Assessing Risk Factors for Cirrhosis-Related Complications of EGVB at Admission

The results of the univariate analysis showed that the early detection of cirrhosis could be challenging and might take longer than the usual diagnosis time among the elderly. Moreover, aged individuals presented an absence of splenectomy, a higher Child-Pugh score, elevated levels of D-D and ALP, lower blood Ca^+2^ level, and a higher neutrophil count. Additionally, higher NLR, WBC, and PLT counts and increased CLIF-C AD scores were associated with a higher risk of complications other than cirrhotic esophagogastric variceal bleeding at admission (odds ratio (OR) = 1.205; *p* < 0.05; Table 2).

### 3.4. Top Three Major Complications and Risk Factors for Postendoscopic COTB

#### 3.4.1. Analysis of Risk Factors for the Occurrence of PVT or Cavernous Lesions

Univariate analysis revealed that the risk of subsequent PVT or cavernous changes was considerably higher in elderly male patients. These patients required a longer diagnosis time and had a history of splenectomy. Notably, these elderly patients without any antiviral therapies exhibited noncholestatic cirrhosis and higher levels of GGT and ALP, along with increased scores of CLIF-C AD. The multifactorial Cox regression analysis indicated that a longer duration of cirrhosis could be a risk factor for subsequent PVT (hazard ratio (HR) = 1.003; *p* < 0.05) and a lower risk factor of PVT and/or cavernous lesions in cholestatic cirrhosis compared to the posthepatitis B cirrhosis (HR = 0.201; *p*=0.041; Table 3).

#### 3.4.2. Analysis of Risk Factors for the Occurrence of Infection

Furthermore, univariate analysis revealed that EGVB patients who had a higher D-D and lower levels of Alb, Ca^+2^, and Hb levels were at the increased risk of subsequent infections. Multifactorial Cox regression analysis also confirmed that severe EGV pathology and a higher D-D level were independent risk factors for secondary infections (HR = 1.093; *p* < 0.05; Table 4).

#### 3.4.3. Analysis of Risk Factors for the Development of Cirrhosis-Related Cholelithiasis

Univariate analysis demonstrated that patients without a history of splenectomy and sclerotherapy, lower blood Ca^+2^ levels, and reduced PLT count had a higher risk of secondary cirrhosis-related cholelithiasis, which was further validated by the multifactorial Cox regression analysis (HR = 0.25; *p* < 0.05; Table 5), indicating that a higher blood Ca^+2^ level could be a protective factor against the subsequent cholelithiasis.

## 4. Discussion

Liver cirrhosis is a chronic liver disease with clinical manifestations of liver scars, portal hypertension, and even serious secondary complications such as viral infections, liver coma, and gastrointestinal bleeding in advanced stages. However, EGVB is the most common complication of liver cirrhosis in the decompensated phase. It has been shown that approximately 30–40% of liver cirrhosis patients exhibit upper gastrointestinal bleeding as the disease progresses, which is often the most common cause of first hospitalization in patients with advanced cirrhosis [22]. Also, several studies have reported that EGVB can complicate the outcome in liver cirrhosis patients [23–25]. These studies have further analyzed the risk factors of bleeding and rebleeding after endoscopic treatment to inhibit the recurrence of EGVB and improve the quality of life of these patients. However, cirrhosis is often combined with other complications in addition to EGVB, which might subside the treatment benefits and prognosis of liver cirrhosis [13–15].

We first retrospectively analyzed the clinical data and found that 361 patients had already presented other complications of cirrhosis at the time of admission. The top three complications were PVT or sponge-like changes, cholelithiasis, and infections. A total of 171 cases did not present COTB in cirrhosis at the time of admission, but bleeding symptoms were prevalent during the follow-up (Table 1), which was consistent with the main complications of cirrhosis described in the Chinese textbook such as bleeding esophageal varices, cholelithiasis, infection, liver encephalopathy, and PVT or spongiform changes. Notably, other studies have reported a wide variety of complications in liver cirrhosis patients, which might be attributed to the different races and geographical locations of the study populations [2, 26, 27]. The three major complications occurring before and after the bleeding treatments were consistent, suggesting that bleeding treatment did not affect these complications.

The COTB at admission can aggravate EGVB and interfere with the first endoscopic treatment [13–15]. Furthermore, the risk factor assessment of these patients showed that the Child-Pugh score was an independent risk factor of COTB at admission. The Child-Pugh scoring is the most used scoring system to assess the prognosis of patients with cirrhosis [28], and it includes indicators that are the major products of liver metabolism such as bilirubin, Alb, and thrombin. A higher Child-Pugh score indicates deteriorating liver functions and an enhanced likelihood of multiple comorbidities [29]. Based on these results, it is imperative to determine the grade of liver function first and design strategies to rescue the liver from irreversible injuries.

Among the 171 patients, COTB in the cirrhotic esophagus did not occur at the time of admission but did during the follow-up or subsequent treatments for bleeding prevention, which possibly exerted a negative impact on the prognosis [16, 17]. The risk factor analysis of the top three complications of this group of patients revealed that the longer diseased period, etiology of cirrhosis, and no prior history of splenectomy were independent predictors of PVT and/or cavernous lesions in these patients. The latter is caused by the long-term effects of portal hypertension in cirrhosis, while portal/cavernous venous thrombosis changes, once formed, exacerbate portal hypertension, leading to a vicious cycle of liver injury. Thus, cirrhosis and cavernous changes in the portal vein can pathologically interact with each other, suggesting that the length of the cirrhotic phase could be directly linked to the onset of secondary complications. Among these causes, cholestatic cirrhosis has a reduced risk of PVT development compared to posthepatitis B cirrhosis. There has been only a handful of studies investigating the crosstalk between biliary cirrhosis and PVT/portal spongiform. One study [30] reported an increased risk of PVT in the patients with autoimmune hepatitis awaiting liver transplantation, which is inconsistent with the findings of the present study. Regarding the underlying etiologies, the low risk of PVT and/or cavernous changes in the patients with biliary cirrhosis has been linked to the medications taken by this group of patients. Patients with cholestatic cirrhosis are usually treated with ursodeoxycholic acid for a long time. These patients often present other systemic symptoms such as rheumatoid arthritis, dry syndrome, thyroiditis, and autoimmune diseases that require treatment with steroid hormones and drugs, which might reduce the regional portal pressure [31, 32]. In this study, 34 of the 36 patients with cholestatic cirrhosis were treated with ursodeoxycholic acid and/or steroid hormones, thus minimizing the incidence of PVT and/or spongiosis. It is discovered through a 2015 meta-analysis [33] that D-dimer concentrations are higher among cirrhotic patients with PVT compared with those without PVT. However, no correlation between D-dimer and portal thrombosis is identified in our study, thus raising our concerns. Related studies have shown that more factors promote portal thrombosis, mainly 3 types of factors: slow portal blood flow, changes in coagulation mechanisms, and endothelial damage [34]. It is difficult to determine the formation of PVT by merely one factor, for it should be verified in several studies. In a retrospective study of 66 patients [35], 24 are diagnosed with PVT, indicating little discrepancy in the D-dimer level between the PVT and non-PVT groups, and there is no correlation between D-dimer and PVT in cirrhosis. Our data encompass 547 cases, of which 178 (49.31%) are combined with PVT and all involve recent bleeding, considering that bleeding decreases the D-dimer levels of patients and thus affects the statistical results of the relationship between D-dimer and portal thrombosis in cirrhosis. As most of the current studies on the association between D-dimer and PVT in cirrhosis are retrospective analyses (mostly data between 2003 and 2010) by nature, there is a lack of prospective and multicentre study data. We have already conducted follow-up prospective multicentre studies (at the First Affiliated Hospital of Anhui Medical University, the 901 Hospital of Joint Logistics Support Force, and Fuyang Second People's Hospital) to continuously follow the relationship between the two.

Here, the degree of EGV and the D-D level was found to be independent risk factors for secondary infections. A higher degree of esophageal varices suggests a higher portal pressure, which in combination with collateral circulation leads to a decrease in the abdominal blood flow entering the liver through the portal vein and affects the detoxification and immune function of the liver. This condition ultimately compromises the defense mechanism of the body, leading to an increased susceptibility to pathogenic infections [36]. Moreover, the degree of portal vein hypertension is positively correlated with the degree of decompensation of cirrhosis. Of note, the severity of liver cirrhosis facilitates the impact on the liver-intestinal axis, resulting in an altered intestinal barrier and increased permeability of pathogens to the blood circulation [37]. The serum level of D-D, as a cross-linked fibrin hydrolysis product, is closely related to the coagulation and fibrinolytic status of the blood. Studies have reported that this factor is significantly elevated in patients with cirrhosis [38, 39] and is associated with PVT pathogenesis [40]. In addition to suggesting the blood coagulation status, the serum D-D level reflects the occurrence of infection, inflammation, and trauma in the body. *In vitro* experiments have demonstrated that D-D can influence the process of immune cell differentiation, which in turn alters the distribution of its downstream inflammatory factors. An increased level of D-D is associated with the activation of the coagulation system under systemic inflammations. One study [41] has shown that the serum D-D level can be used as a diagnostic marker for spontaneous peritonitis in cirrhosis, which is consistent with the results of the present study.

In patients with cirrhosis, hypocalcemia is prevalent due to reduced intake, poor absorption, low Alb, decreased parathyroid hormone, and reduced vitamin D levels [42]. Blood calcium concentration can be used as an indicator to determine the degree of impairment of the liver function in patients with cirrhosis. It also has clinical significance and a predictive value for the evaluation of the treatment and prognosis of liver cirrhosis [43]. Studies [44] have reported that elevated blood calcium is a critical risk factor for cirrhosis, but the mechanism remains yet unknown. We found that low blood calcium levels could be an independent risk factor for cirrhosis-related gallstone diseases. Since no relevant studies have been reported for reference, the specific mechanism is still unclear. It might be explained as follows: long-term hypocalcemia in liver cirrhosis patients can lead to disturbances in calcium homeostasis, altered bile acid secretion, and gallbladder contraction dysfunction, facilitating the formation and deposition of insoluble calcium salts upon reacting with bilirubin to form gallbladder stones. Therefore, further studies are warranted to better understand this mechanism.

In conclusion, the higher Child-Pugh score in liver cirrhosis patients undergoing endoscopic treatments for EGVB is likely to induce multiple concurrent comorbidities. Furthermore, a relatively longer duration of cirrhosis can significantly increase the probability of sequential PVT. However, cholestatic cirrhosis has a lower risk of PVT or cavernous changes than patients with posthepatitis B cirrhosis. Moreover, patients with advanced-stage EGV and elevated levels of D-D in the serum could have increased susceptibility towards pathogenic infections. Besides, lower blood calcium levels may increase the risk of cholelithiasis. Clinically, patients can receive targeted treatments and interventions accordingly to avoid the onset of other secondary combinations during and after the treatment of EGVB in cirrhosis, thus improving the outcome and prognosis of the patients suffering from bleeding symptoms.

## 5. Limitations

This is a single-center clinical retrospective study, and the conclusions only represent the disease characteristics of the patients in our center. The universality of the conclusions needs further support from multicentre studies.

## Figures and Tables

**Table 1:** Complications of cirrhosis other than esophagogastric variceal bleeding at admission and at the late follow-up.

Other complications of cirrhosis at the time of admission	Number and proportion of complications (361 cases)	Other complications of cirrhosis occur later in life	Total number and proportion of complications (171 cases)
Portal vein thrombosis and/or cavernous lesions	178 (49.31%)	Portal vein thrombosis and/or cavernous lesions	89 (52.05%)
Gallstone disease	152 (42.11%)	Infection	70 (40.94%)
Infection	101 (27.98%)	Gallstone disease	68 (39.77%)
Primary liver cancer	33 (9.14%)	Electrolyte disorders	47 (27.49%)
Electrolyte disorders	19 (5.26%)	Hepatic encephalopathy	23 (13.45%)
Hepatic encephalopathy	8 (2.22%)	Primary liver cancer	23 (13.45%)
Hepatorenal syndrome	2 (0.55%)	Hepatorenal syndrome	6 (3.51%)
Hepatopulmonary syndrome	0	Hepatopulmonary syndrome	1 (0.58%)

*Note.* Some patients had a combination of two or more complications.

**Table 2:** Univariate and multivariate analyses of risk factors for patients with extra-hemorrhagic complications at admission (logistic regression).

Characteristics	Single factor analysis			Multifactor analysis		
	OR	95% CI	*p*	OR	95% CI	*p*
Gender (female)	1.13	0.78–1.66	0.52			
Age (years old)	1.02	1.01–1.035	0.011	1.039	0.942–1.145	0.444
Time of cirrhosis was found (years)	1	1–1.01	0.03	1.002	0.998–1.005	0.411
BMI	0.972	0.94–1.005	0.121			
Blood pressure	0.67	0.44–1	0.051			
Heart rate	1.26	0.75–2.11	0.38			
Liver and palm spider nevus (yes)	1.03	0.65–1.63	0.89			
History of splenectomy (yes)	0.318	0.171–0.593	<0.001	0.392	0.153–1.005	0.051
Education level primary school vs. none	0.7	0.36–1.38	0.3			
Education level: junior high vs. none	0.61	0.31–1.21	0.16			
Education level: high school vs. none	0.68	0.32–1.44	0.31			
University education or above vs. none	0.52	0.14–1.89	0.32			
Smoking (yes)	1.13	0.73–1.74	0.59			
Drink alcohol (yes)	1.19	0.81–1.74	0.38			
Antiviral drug use. No vs. yes	0.94	0.64–1.36	0.73			
Whether to take beta blockers. No vs. yes	0.52	0.11–2.54	0.42			
Etiology of cirrhosis: Posthepatitic C vs. posthepatitic B	2.04	0.42–9.99	0.38			
Etiology of cirrhosis. Alcoholic cirrhosis vs. posthepatitis B cirrhosis	0.85	0.47–1.53	0.58			
Etiology of cirrhosis. Mixed cirrhosis vs. posthepatitis B cirrhosis	2.11	0.93–4.79	0.07			
Etiology of cirrhosis. Autoimmune cirrhosis vs. posthepatitis B cirrhosis	0.65	0.24–1.75	0.4			
Etiology of cirrhosis: Cholestatic cirrhosis vs. posthepatitis B cirrhosis	2.04	0.89–4.64	0.09			
Schistosomiasis is the cause of cirrhosis	0.58	0.16–2.06	0.4			
Etiology of cirrhosis. Unknown cause vs. posthepatitis B cirrhosis	1.11	0.69–1.79	0.66			
Diabetes (yes)	0.88	0.55–1.39	0.58			
HBV titer PCR.IUML	0.71	0.39–1.29	0.27			
Degree of esophageal and fundus varices. Heavy vs. medium	1.157	0.691–1.936	0.58			
The treatment of varicose veins. Sclerotherapy vs. ligation	1.17	0.75–1.82	0.49			
The treatment of varicose veins. Tissue glue vs. ligation	1.4	0.48–4.07	0.53			
The treatment of varicose veins. Combination therapy vs. ligation	0.87	0.56–1.35	0.55			
Grading of child	1.16	1.06–1.26	<0.001	1.204	1.068–1.357	0.002
Whether it is complicated with liver cancer (yes)	0.75	0.4–1.42	0.38			
Portal hypertensive gastropathy (yes)	1.43	0.82–2.51	0.21			
D-dimer	1.22	1.09–1.36	<0.001	1.13	1–1.281	0.051
International standardized ratio (INR)	1.87	0.81–4.34	0.15			
Prothrombin time (PT)	1	0.96–1.03	0.82			
Alanine aminotransferase (ALT)	1	1–1.01	0.18			
Aspartic acid aminotransferase (AST)	1	1–1	0.14			
Aspartate aminotransferase. (AST), platelets (PLT), APRI	1.15	0.98–1.36	0.09			
Albumin (ALB)	0.96	0.93–0.98	0.003	1.016	0.962–1.073	0.563
*γ* Glutamine transferase. Gamma GT	1	1–1	0.81			
Alkaline phosphatase (ALP)	1	1–1.01	0.01	1.001	0.999–1.004	0.278
Total bilirubin (TBil)	1.01	1–1.02	0.26			
Creatinine (Cr)	1	0.99–1.01	0.8			
The urea	1.06	1–1.13	0.06			
Glomerular filtration rate (GFR)	1	0.99–1.01	0.84			
Triglyceride trifat	1.03	0.95–1.11	0.47			
Cholesterol	0.89	0.72–1.1	0.29			
Blood calcium	0.3	0.1–0.92	0.04	0.594	0.101–3.499	0.565
Serum sodium	0.97	0.93–1.02	0.27			
Neutrophils (Neu)	1.18	1.06–1.31	<0.001	0.8	0.424–1.507	0.489
Lymphocyte count (LYM)	0.98	0.85–1.13	0.75			
Neutrophils. Ratio of neutrophils to lymphocytes	1.14	1.05–1.24	0.002	1.189	0.999–1.416	0.052
White blood cell (WBC) count	1.14	1.05–1.23	<0.001	1.181	0.733–1.902	0.494
Hemoglobin (Hb)	0.99	0.99–1	0.07			
Platelet count (PLT)	1	1–1.01	0.01	1.001	0.997–1.005	0.604
Erythrocyte distribution width. RDW. CV	1.06	0.99–1.13	0.08			
CLIF-C Ads	1.076	1.025–1.131	0.003	0.923	0.662–1.286	0.636

CLIF-C Ads, CLIF consortium acute decompensation score; OR, odds ratio; CI, confidence interval.

**Table 3:** Univariate and multivariate analyses of factors affecting the occurrence of portal vein thrombosis and/or cavernous lesions in liver cirrhosis patients during the late follow-up (Cox regression analysis).

Characteristics	Single-factor analysis			Multifactor analysis		
	HR	95% CI	*p*	HR	95% CI	*p*
Gender: 1 male and 2 female	0.52	0.31–0.89	0.017	0.884	0.444–1.76	0.725
Age (years old)	0.973	0.954–0.992	0.006	1.072	0.644–1.784	0.79
Time of cirrhosis was found (years)	1.004	1.001–1.006	0.005	1.004	1.001–1.007	0.022
The square of the height of the kg. weight	1.035	0.993–1.079	0.103			
Blood pressure. Systolic pressure 90–139 vs. 90	2.03	0.64–6.45	0.229			
Blood pressure. Systolic blood pressure 140–159 vs. 90	2.07	0.57–7.54	0.269			
Blood pressure. Systolic blood pressure 160–179 vs. 90	0	0-Inf	0.997			
Blood pressure. Systolic blood pressure greater than 180 vs. 90	0	0-Inf	0.998			
Heart rate. 60–100 beats per minute vs. less than 60 beats per minute	2.8	0.39–20.14	0.306			
Heart rate. Over 100 beats per minute vs. less than 60 beats per minute	4.47	0.57–34.89	0.154			
Spider nevus of liver and palm (none)	1.02	0.62–1.67	0.949			
History of splenectomy (none)	0.62	0.38–1	0.049	1.024	0.58–1.808	0.935
Education level primary school vs. none	1.22	0.56–2.67	0.62			
Education level: junior high vs. none	1.69	0.77–3.7	0.192			
Education level: high school vs. none	1.94	0.86–4.4	0.111			
University education or above vs. none	1.56	0.33–7.37	0.576			
Smoking (yes)	0.99	0.6–1.64	0.965			
Drink alcohol (yes)	0.98	0.63–1.53	0.932			
Antiviral drug use. No vs. yes	0.595	0.391–0.907	0.016	0.917	0.533–1.576	0.753
Whether to take beta blockers. No vs. yes	1.1	0.27–4.46	0.897			
Etiology of cirrhosis: posthepatitic C vs. posthepatitic B	0.51	0.12–2.09	0.349	1.11	0.248–4.964	0.891
Etiology of cirrhosis. Alcoholic cirrhosis vs. posthepatitis B cirrhosis	0.41	0.18–0.96	0.045	0.63	0.239–1.662	0.35
Etiology of cirrhosis. Mixed cirrhosis vs. posthepatitis B cirrhosis	0.72	0.36–1.47	0.37	0.779	0.347–1.748	0.545
Etiology of cirrhosis. Autoimmune cirrhosis. vs. posthepatitis B cirrhosis	0.38	0.09–1.57	0.181	0.563	0.114–2.788	0.482
Etiology of cirrhosis: cholestatic cirrhosis vs. posthepatitis B cirrhosis	0.12	0.03–0.51	0.004	0.201	0.043–0.938	0.041
Schistosomiasis is the cause of cirrhosis	0	0-Inf	0.995	0	0–1.8 E+271	0.973
Etiology of cirrhosis. Unknown cause vs. posthepatitis B cirrhosis	1.02	0.56–1.83	0.958	1.102	0.537–2.264	0.791
Diabetes (yes)	1.44	0.8–2.59	0.225			
HBV titer PCR.IUML. 1. Negative. 1000ium.2. Positive. 1000 IUml.l	0.79	0.35–1.82	0.583			
Degree of esophageal and fundus varices. Heavy vs. medium	0.699	0.431–1.132	0.146			
The treatment of varicose veins. Sclerotherapy vs. ligation	0.91	0.55–1.48	0.694			
The treatment of varicose veins. Tissue glue vs. ligation	1.52	0.47–4.93	0.486			
The treatment of varicose veins. Combination therapy vs. ligation	1.22	0.7–2.11	0.486			
Grading of child	0.97	0.89–1.05	0.438			
Whether it is complicated with liver cancer (1) yes and (2) no	0.92	0.4–2.08	0.836			
Whether there is portal hypertensive gastropathy (1) yes and (2) no	0.84	0.42–1.68	0.63			
D-dimer	1.02	0.95–1.09	0.615			
International standardized ratio (INR)	1.1	0.5–2.42	0.805			
Prothrombin time (PT)	1.02	0.95–1.1	0.531			
Alanine aminotransferase (ALT)	1	1-1	0.758			
Aspartic acid aminotransferase (AST)	1	44562	0.358			
Aspartate aminotransferase (AST). Platelets (PLT). APRI	0.95	0.82–1.09	0.47			
Albumin (ALB)	1.01	0.98–1.03	0.568			
Gamma transglutaminase	0.997	0.994–1	0.033	0.998	0.993–1.002	0.301
Alkaline phosphatase (ALP)	0.997	0.994–1	0.022	1	0.995–1.005	0.911
Total bilirubin (TBil)	0.99	0.97–1	0.052			
Creatinine (Cr)	1	0.99–1	0.335			
The urea	0.99	0.93–1.04	0.635			
Glomerular filtration rate (GFR)	1.01	1–1.02	0.103			
Triglyceride trifat	0.53	0.24–1.19	0.125			
Cholesterol	0.99	0.82–1.19	0.886			
Blood calcium	1.17	0.35–3.89	0.8			
Serum sodium	1	0.93–1.07	0.937			
Neutrophils (Neu)	0.95	0.86–1.05	0.306			
Lymphocyte count (LYM)	0.97	0.73–1.29	0.846			
Neutrophils. Ratio of neutrophils to lymphocytes	0.97	0.91–1.04	0.422			
White blood cell count	0.97	0.91–1.04	0.412			
Hemoglobin (Hb)	1	1–1.01	0.269			
Platelet count (PLT)	1	1–1	0.15			
Erythrocyte distribution width. RDW. CV	0.99	0.92–1.07	0.781			
CLIF-C Ads	0.93	0.881–0.981	0.008	0.954	0.898–1.014	0.13

CLIF-C Ads, CLIF consortium acute decompensation score; HR, hazard ratio; CI, confidence interval.

**Table 4:** Univariate and multivariate analyses of factors affecting the occurrence of infection in liver cirrhosis patients during the late follow-up (Cox regression analysis).

Characteristics	Single-factor analysis			Multifactor analysis		
	HR	95% CI	*p*	HR	95% CI	*p*
Gender: 1 male and 2 female	1.04	0.62–1.75	0.88			
Age (years old)	1.02	1–1.04	0.09			
Time of cirrhosis was found (years)	1	1-1	0.685			
The square of the height of the kg weight	1.2	0.6–2.38	0.606			
Blood pressure. Systolic pressure 90–139 vs. 90	0.73	0.29–1.83	0.508			
Blood pressure. Systolic blood pressure 140–159 vs. 90	0.56	0.17–1.84	0.338			
Blood pressure. Systolic blood pressure 160–179 vs. 90	1.53	0.29–7.91	0.614			
Blood pressure. Systolic blood pressure greater than 180 vs. 90	0	0-Inf	0.995			
Heart rate. 60–100 beats per minute vs. less than 60 beats per minute	1.13	0.28–4.62	0.866			
Heart rate. Over 100 beats per minute vs. less than 60 beats per minute	0.67	0.12–3.65	0.642			
Liver and palm spider nevus (yes)	0.85	0.5–1.45	0.543			
History of splenectomy (yes)	0.79	0.45–1.38	0.407			
Education level: primary school vs. none	1.12	0.5–2.49	0.78			
Education level: junior high vs. none	1.4	0.63–3.11	0.414			
Education level high school vs. none	0.67	0.26–1.74	0.407			
University education or above vs. none	0	0-Inf	0.995			
Smoking (yes)	1.34	0.72–2.5	0.355			
Drink alcohol (yes)	0.89	0.54–1.46	0.636			
Antiviral drug use. No vs. yes	1.06	0.65–1.73	0.817			
Whether to take beta blockers. No vs. yes	1.99	0.28–14.41	0.494			
Etiology of cirrhosis: posthepatitic C vs. posthepatitic B	2	0.61–6.57	0.253			
Etiology of cirrhosis. Alcoholic cirrhosis vs. posthepatitis B cirrhosis	1.67	0.79–3.52	0.177			
Etiology of cirrhosis. Mixed cirrhosis vs. posthepatitis B cirrhosis	1.41	0.65–3.07	0.39			
Etiology of cirrhosis. Autoimmune cirrhosis. vs. posthepatitis B cirrhosis	1.9	0.67–5.41	0.227			
Etiology of cirrhosis: cholestatic cirrhosis vs. posthepatitis B cirrhosis	1.73	0.79–3.77	0.17			
Schistosomiasis is the cause of cirrhosis	0	0-Inf	0.995			
Etiology of cirrhosis. Unknown cause vs. posthepatitis B cirrhosis	1.02	0.47–2.24	0.951			
Diabetes (yes)	0.86	0.48–1.55	0.621			
HBV titer PCR.IUML. 1. Negative. 1000ium.2. Positive.1000 IUml.l	0.93	0.37–2.32	0.881			
Degree of esophageal and fundus varices. Heavy vs. medium	0.24	0.08–0.77	0.016	0.203	0.062–0.667	0.009
The treatment of varicose veins. Sclerotherapy vs. ligation	0.79	0.44–1.4	0.414			
The treatment of varicose veins. Tissue glue vs. ligation	1.1	0.26–4.61	0.897			
The treatment of varicose veins. Combination therapy vs. ligation	1.27	0.69–2.33	0.44			
Grading of child	1.1	0.99–1.22	0.08			
Whether it is complicated with liver cancer. (1) yes and (2) no	0.93	0.41–2.13	0.867			
Whether there is portal hypertensive gastropathy (1) yes and (2) no	1.69	0.61–4.63	0.31			
D-dimer	1.09	1.01–1.17	0.025	1.093	1.002–1.193	0.046
International standardized ratio (INR)	1.27	0.5–3.24	0.612			
Prothrombin time (PT)	1.04	0.96–1.12	0.371			
Alanine aminotransferase (ALT)	1	1-1	0.553			
Aspartic acid aminotransferase (AST)	1	1-1	0.495			
Aspartate aminotransferase (AST). Platelets (PLT). APRI	0.93	0.79–1.11	0.443			
Albumin (ALB)	0.96	0.92–0.99	0.022	0.979	0.932–1.03	0.416
Gamma transglutaminase	1	1-1	0.715			
Alkaline phosphatase (ALP)	1	1-1	0.407			
Total bilirubin (TBil)	1	0.98–1.01	0.841			
Creatinine (Cr)	1	0.99–1.01	0.487			
The urea	1.04	0.99–1.09	0.127			
Glomerular filtration rate (GFR)	1	0.99–1.01	0.744			
Triglyceride trifat	1	0.37–2.73	0.998			
Cholesterol	0.85	0.61–1.2	0.355			
Blood calcium	0.18	0.08–0.43	<0.001	0.497	0.117–2.11	0.343
Serum sodium	1.06	0.97–1.15	0.184			
Neutrophils (Neu)	1.01	0.93–1.09	0.866			
Lymphocyte count (LYM)	0.97	0.7–1.34	0.85			
Neutrophils. Ratio of neutrophils to lymphocytes	1.01	0.96–1.06	0.78			
White blood cell count	1	0.94–1.07	0.948			
Hemoglobin (Hb)	0.99	0.98–1	0.047	0.997	0.983–1.011	0.997
Platelet count (PLT)	1	1-1	0.731			
Erythrocyte distribution width. RDW. CV	1.04	0.96–1.13	0.315			
CLIF-C Ads	1.043	0.976–1.114	0.21			

CLIF-C Ads, CLIF consortium acute decompensation score; HR, hazard ratio; CI, confidence interval.

**Table 5:** Univariate and multivariate analyses of factors affecting the occurrence of cholelithiasis in liver cirrhosis patients during the late follow-up (Cox regression analysis).

Characteristics	Single-factor analysis			Multifactor analysis		
	HR	95% CI	*p*	HR	95% CI	*p*
Gender: 1 male and 2 female	1.29	0.78–2.15	0.321			
Age (years old)	1.01	0.99–1.03	0.506			
Time of cirrhosis was found (years)	1	1–1.01	0.154			
The square of the height of the kg weight	1.015	0.967–1.066	0.546			
Blood pressure. Systolic pressure 90–139 vs. 90	1.27	0.4–4.06	0.683			
Blood pressure. Systolic blood pressure 140–159 vs. 90	0.8	0.19–3.36	0.763			
Blood pressure. Systolic pressure 160–179 vs. 90	1.31	0.14–12.61	0.815			
Blood pressure. Systolic blood pressure greater than 180 vs. 90	0	0-Inf	0.995			
Heart rate. 60–100 beats per minute vs. less than 60 beats per minute	24892341.78	0-Inf	0.996			
Heart rate. Over 100 beats per minute vs. less than 60 beats per minute	36261614.52	0-Inf	0.996			
Spider nevus of liver and palm (none)	1.05	0.59–1.86	0.87			
History of splenectomy (none)	2.44	1.12–5.35	0.025	1.42	0.54–3.71	0.4783
Education level primary school vs. none	0.88	0.42–1.87	0.741			
Education level: junior high vs. none	0.69	0.31–1.52	0.351			
Education level: high school vs. none	0.56	0.23–1.38	0.209			
University education or above vs. none	1.34	0.29–6.21	0.707			
Smoking (yes)	1.1	0.6–2.01	0.757			
Drink alcohol (yes)	1.75	0.99–3.11	0.055			
Antiviral drug use. No vs. yes	1.1	0.67–1.82	0.708			
Whether to take beta blockers. No vs. yes	2.02	0.28–14.54	0.486			
Etiology of cirrhosis: Posthepatitic C vs. posthepatitic B	1.45	0.45–4.69	0.537			
Etiology of cirrhosis. Alcoholic cirrhosis. vs. posthepatitis B cirrhosis	0.54	0.21–1.36	0.191			
Etiology of cirrhosis. Mixed cirrhosis vs. posthepatitis B cirrhosis	0.41	0.15–1.16	0.093			
Etiology of cirrhosis. Autoimmune cirrhosis. vs. posthepatitis B cirrhosis	1.06	0.33–3.42	0.926			
Etiology of cirrhosis: Cholestatic cirrhosis vs. posthepatitis B cirrhosis	0.96	0.43–2.14	0.913			
Schistosomiasis is the cause of cirrhosis	0	0-Inf	0.995			
Etiology of cirrhosis. Unknown cause vs. posthepatitis B cirrhosis	0.58	0.26–1.3	0.184			
Diabetes (yes)	1.35	0.69–2.63	0.386			
HBV titer PCR.IUML. 1. Negative. 1000 IUml.2. Positive.1000 IUml.l	1.54	0.7–3.36	0.283			
Degree of esophageal and fundus varices. Heavy vs. medium	1.184	0.634–2.21	0.621			
The treatment of varicose veins. Sclerotherapy vs. ligation	0.7	0.4–1.23	0.21	0.7	0.39–1.27	0.2448
The treatment of varicose veins. Tissue glue vs. ligation	3.09	1.09–8.78	0.034	2.8	0.98–8.03	0.055
The treatment of varicose veins. Combination therapy vs. ligation	0.79	0.41–1.53	0.479	0.65	0.32–1.33	0.2369
Grading of child	1.02	0.92–1.13	0.653			
Whether it is complicated with liver cancer. (1) yes and (2) no	1.53	0.51–4.55	0.446			
Whether there is portal hypertensive gastropathy. (1) yes and (2) no	0.8	0.36–1.74	0.566			
D-dimer	0.95	0.85–1.05	0.286			
International standardized ratio (INR)	1.23	0.49–3.09	0.655			
Prothrombin time (PT)	1.02	0.94–1.11	0.606			
Alanine aminotransferase (ALT)	1	1-1	0.789			
Aspartic acid aminotransferase (AST)	1	1-1	0.195			
Aspartate aminotransferase (AST). Platelets (PLT). APRI	1.09	1–1.19	0.059			
Albumin (ALB)	0.99	0.96–1.02	0.587			
Gamma transglutaminase	1	1-1	0.756			
Alkaline phosphatase (ALP)	1	1-1	0.427			
Total bilirubin (TBil)	1	0.99–1.01	0.901			
Creatinine (Cr)	1	1–1.01	0.323			
The urea	1.04	0.99–1.09	0.089			
Glomerular filtration rate (GFR)	1	0.99–1.01	0.591			
Triglyceride trifat	1.62	0.73–3.58	0.234			
Cholesterol	1.03	0.8–1.32	0.834			
Blood calcium	0.23	0.06–0.82	0.023	0.25	0.07–0.95	0.0425
Serum sodium	1.06	0.97–1.15	0.205			
Neutrophils (Neu)	1.07	0.99–1.15	0.083			
Lymphocyte count. LYM	0.69	0.47–1.03	0.066			
Neutrophils. Ratio of neutrophils to lymphocytes	1.04	0.99–1.08	0.124			
White blood cell count	1.02	0.95–1.1	0.559			
Hemoglobin (Hb)	0.99	0.99–1	0.312			
Platelet count (PLT)	0.99	0.99–1	0.005	1	0.99–1	0.1251
Erythrocyte distribution width. RDW. CV	1	0.92–1.1	0.927			
CLIF-C Ads	1.037	0.984–1.093	0.172			

CLIF-C Ads, CLIF consortium acute decompensation score; HR, hazard ratio; CI, confidence interval.

## Data Availability

The datasets used in this study are available on reasonable request to the corresponding author.

## References

[1] Tapper E. B., Parikh N. D. (2018). Mortality due to cirrhosis and liver cancer in the United States, 1999-2016: observational study. *BMJ*.

[2] Jang W. Y., Chung W. J., Jang B. K. (2020). Changes in characteristics of patients with liver cirrhosis visiting a tertiary hospital over 15 years: a retrospective multi-center study in korea. *Journal of Korean Medical Science*.

[3] Duah A., Duah F., Ampofo-Boobi D., Addo B. P., Osei-Poku F., Agyei-Nkansah A. (2022). Acute kidney injury in patients with liver cirrhosis: prevalence, predictors, and in-hospital mortality at a district hospital in Ghana. *BioMed Research International*.

[4] Ginès P., Krag A., Abraldes J. G., Solà E., Fabrellas N., Kamath P. S. (2021). Liver cirrhosis. *The Lancet*.

[5] de Franchis R., Baveno V. F. (2010). Revising consensus in portal hypertension: report of the Baveno V consensus workshop on methodology of diagnosis and therapy in portal hypertension. *Journal of Hepatology*.

[6] Lesmana C. R. A., Raharjo M., Gani R. A. (2020). Managing liver cirrhotic complications: overview of esophageal and gastric varices. *Clinical and Molecular Hepatology*.

[7] Ponnusamy R., Cherian J., Deepak N., Somasundaram A., Jayanthi V. (2011). Non-invasive predictors of esophageal varices. *Saudi Journal of Gastroenterology*.

[8] Tsochatzis E. A., Bosch J., Burroughs A. K. (2014). Liver cirrhosis. *The Lancet*.

[9] Hwang J. H., Shergill A. K., Acosta R. D. (2014). The role of endoscopy in the management of variceal hemorrhage. *Gastrointestinal Endoscopy*.

[10] de Franchis R., VI Faculty B. (2015). Expanding consensus in portal hypertension: report of the Baveno VI Consensus Workshop: stratifying risk and individualizing care for portal hypertension. *Journal of Hepatology*.

[11] Qi X., Li J., Han B. (2018). Association of coagulopathy with the risk of bleeding after invasive procedures in liver cirrhosis. *Saudi Journal of Gastroenterology*.

[12] Tantai X. X., Liu N., Yang L. B. (2019). Prognostic value of risk scoring systems for cirrhotic patients with variceal bleeding. *World Journal of Gastroenterology*.

[13] Deschênes M., Villeneuve J. P. (1999). Risk factors for the development of bacterial infections in hospitalized patients with cirrhosis. *American Journal of Gastroenterology*.

[14] El Sheref S. E. D. M., Afify S., Berengy M. S. (2022). Clinical characteristics and predictors of esophagogastric variceal bleeding among patients with HCV-induced liver cirrhosis: an observational comparative study. *PLoS One*.

[15] Zhou H., Xuan J., Lin X., Guo Y. (2018). Recurrent esophagogastric variceal bleeding due to portal vein thrombosis caused by protein S deficiency. *Endoscopy International Open*.

[16] Satapathy S. K., Sanyal A. J. (2014). Nonendoscopic management strategies for acute esophagogastric variceal bleeding. *Gastroenterology Clinics of North America*.

[17] Bernard B., Cadranel J. F., Valla D., Escolano S., Jarlier V., Opolon P. (1995). Prognostic significance of bacterial infection in bleeding cirrhotic patients: a prospective study. *Gastroenterology*.

[18] Garcia-Tsao G., Sanyal A. J., Grace N. D., Carey W. D. (2007). Prevention and management of gastroesophageal varices and variceal hemorrhage in cirrhosis. *American Journal of Gastroenterology*.

[19] Wang H., Wen B., Chang X. (2021). Baveno VI criteria and spleen stiffness measurement rule out high-risk varices in virally suppressed HBV-related cirrhosis. *Journal of Hepatology*.

[20] Zhao Y., Ren M., Lu G. (2020). The prognosis analysis of liver cirrhosis with acute variceal bleeding and validation of current prognostic models: a large scale retrospective cohort study. *BioMed Research International*.

[21] Gross M. (2015). Wenn die Leber den Dienst versagt [Liver cirrhosis and the most common complications: diagnosis and treatment]. *MMW - Fortschritte der Medizin*.

[22] Merchante N., Rivero-Juárez A., Téllez F. (2017). Liver stiffness predicts variceal bleeding in HIV/HCV-coinfected patients with compensated cirrhosis. *AIDS*.

[23] Dell’era A., Bosch J. (2004). Review article: the relevance of portal pressure and other risk factors in acute gastro-oesophageal variceal bleeding. *Alimentary Pharmacology & Therapeutics*.

[24] Pfisterer N., Unger L. W., Reiberger T. (2021). Clinical algorithms for the prevention of variceal bleeding and rebleeding in patients with liver cirrhosis. *World Journal of Hepatology*.

[25] Plaz Torres M. C., Best L. M., Freeman S. C. (2021). Secondary prevention of variceal bleeding in adults with previous oesophageal variceal bleeding due to decompensated liver cirrhosis: a network meta-analysis. *Cochrane Database of Systematic Reviews*.

[26] Nilsson E., Anderson H., Sargenti K., Lindgren S., Prytz H. (2016). Incidence, clinical presentation and mortality of liver cirrhosis in Southern Sweden: a 10-year population-based study. *Alimentary Pharmacology & Therapeutics*.

[27] Abu-Freha N., Estis-Deaton A., Aasla M. (2022). Liver cirrhosis in elderly patients: clinical characteristics, complications, and survival-analyses from a large retrospective study. *Aging Clinical and Experimental Research*.

[28] Peng Y., Qi X., Dai J., Li H., Guo X. (2015). Child-Pugh versus MELD score for predicting the in-hospital mortality of acute upper gastrointestinal bleeding in liver cirrhosis. *International Journal of Clinical and Experimental Medicine*.

[29] Peng Y., Qi X., Guo X. (2016). Child-Pugh versus MELD Score for the assessment of prognosis in liver cirrhosis: a systematic review and meta-analysis of observational studies. *Medicine (Baltimore)*.

[30] Ruiz P., Sastre L., Crespo G. (2017). Increased risk of portal vein thrombosis in patients with autoimmune hepatitis on the liver transplantation waiting list. *Clinical Transplantation*.

[31] Sakamoto Y., Ishigaki K., Ishikawa C., Nakayama T., Asano K., Sakai M. (2020). Successful management of portal vein thrombosis in a Yorkshire Terrier with protein-losing enteropathy. *BMC Veterinary Research*.

[32] Garcia-Pagán J. C., Hernández-Guerra M., Bosch J. (2008). Extrahepatic portal vein thrombosis. *Seminars in Liver Disease*.

[33] Dai J., Qi X., Li H., Guo X. (2015). Role of D-dimer in the development of portal vein thrombosis in liver cirrhosis: a meta-analysis. *Saudi Journal of Gastroenterology*.

[34] Anton A., Campreciós G., Pérez-Campuzano V., Orts L., García-Pagán J. C., Hernández-Gea V. (2022). The pathophysiology of portal vein thrombosis in cirrhosis: getting deeper into virchow’s triad. *Journal of Clinical Medicine*.

[35] Dai J., Qi X., Peng Y. (2015). Association between D-dimer level and portal venous system thrombosis in liver cirrhosis: a retrospective observational study. *International Journal of Clinical and Experimental Medicine*.

[36] Trebicka J., Reiberger T., Laleman W. (2018). Gut-liver Axis links portal hypertension to acute-on-chronic liver failure. *Visual Medicine*.

[37] Jalan R., Fernandez J., Wiest R. (2014). Bacterial infections in cirrhosis: a position statement based on the EASL Special Conference 2013. *Journal of Hepatology*.

[38] Gram J., Duscha H., Zurborn K. H., Bruhn H. D. (1991). Increased levels of fibrinolysis reaction products (D-dimer) in patients with decompensated alcoholic liver cirrhosis. *Scandinavian Journal of Gastroenterology*.

[39] Cioni G., Cristani A., Mussini C. (1990). Incidence and clinical significance of elevated fibrin(ogen) degradation product and/or D-dimer levels in liver cirrhosis patients. *Italian Journal of Gastroenterology*.

[40] Dai J., Qi X., Li H., Guo X. (2015). Role of D-dimer in the development of portal vein thrombosis in liver cirrhosis: a meta-analysis. *Saudi Journal of Gastroenterology*.

[41] El Gohary A. M., Elyamany A. S., Mikhael N. L., Mahmoud M. G., Tawfik M. M. R. (2021). Serum and ascitic D-dimer in cirrhotic patients with spontaneous bacterial peritonitis. *Clinical and Experimental Hepatology*.

[42] Zhao X. Y., Li J., Wang J. H. (2016). Vitamin D serum level is associated with Child-Pugh score and metabolic enzyme imbalances, but not viral load in chronic hepatitis B patients. *Medicine (Baltimore)*.

[43] Hakami Y., Khan A. (2019). Parathyroid Disorders. Focusing on Unmet Needs. *Frontiers of Hormone Research*.

[44] Yin L. Y., Yin J., Cui J. F. (2013). Association between serum calcium levels and the risk of liver cirrhosis. *Zhonghua Liuxingbingxue Zazhi*.

